# Suppression of the NTS-CPS1 regulatory axis by AFF1 in lung adenocarcinoma cells

**DOI:** 10.1016/j.jbc.2021.100319

**Published:** 2021-01-22

**Authors:** Junjie Yue, Qian Dai, Shaohua Hao, Shiqi Zhu, Xiaoxu Liu, Zhiqun Tang, Meng Li, Haitong Fang, Chengqi Lin, Zhuojuan Luo

**Affiliations:** 1 The Key Laboratory of Developmental Genes and Human Disease, School of Life Science and Technology, Southeast University, Nanjing, China; 2 Singapore Eye research Institute, Singapore, Singapore; 3 Co-innovation Center of Neuroregeneration, Nantong University, Nantong, China

**Keywords:** AFF1, lung adenocarcinoma, neurotensin, enhancer, transcription regulation, ChIP-seq, chromatin immunoprecipitation sequencing, CNS, central nervous system, CPS1, carbamoyl phosphate synthetase 1, EGFR, epidermal growth factor receptor, GI, gastrointestinal, GWAS, genome-wide association studies, IL6, interleukin-6, K-M, Kaplan–Meier, NSCLC, non-small-cell lung cancer, NTS, neurotensin, PCR, polymerase chain reaction, SEC, super elongation complex, sgRNA, short guide RNA

## Abstract

Upregulation of the neuropeptide neurotensin (NTS) in a subgroup of lung cancers has been linked to poor prognosis. However, the regulatory pathway centered on NTS in lung cancer remains unclear. Here we identified the NTS-specific enhancer in lung adenocarcinoma cells. The AF4/FMR2 (AFF) family protein AFF1 occupies the NTS enhancer and inhibits NTS transcription. Clustering analysis of lung adenocarcinoma gene expression data demonstrated that NTS expression is highly positively correlated with the expression of the oncogenic factor CPS1. Detailed analyses demonstrated that the IL6 pathway antagonizes NTS in regulating CPS1. Thus, our analyses revealed a novel NTS-centered regulatory axis, consisting of AFF1 as a master transcription suppressor and IL6 as an antagonist in lung adenocarcinoma cells.

Neurotensin (NTS) is a 13 amino acid neuropeptide predominantly found in the central nervous system (CNS) and the gastrointestinal (GI) tract (1, 2, 3). NTS is synthesized as an inactive precursor and subsequently activated by proteolytic cleavage (4, 5). In addition to its physiological roles in neural transmission and digestive processes, NTS is also highly associated with various diseases, including cancers (6, 7, 8, 9). High levels of NTS and its receptors have been implicated in cancer neuroendocrine differentiation and malignant progression (10). It has been demonstrated that NTS can activate the cytokine interleukin-8 (IL-8) pathway in pancreatic and colorectal cancer cells, promoting tumor progression (11). Upregulation of NTS has been observed in a subgroup of lung cancers and linked to poor prognosis (12, 13, 14). However, an integrated understanding about the regulatory pathway centered on NTS in lung cancer remains poorly elucidated.

AFF1 (AF4/FMR2 Family Member 1) is known as the central factor of the super elongation complex (SEC) (15, 16). AFF1 plays pivotal roles in disease pathogenesis and development *via* its transcriptional regulatory activity (17, 18, 19). *AFF1* is frequently fused with *MLL* (Mixed Lineage Leukemia) *via* chromosomal translocation, giving rise to MLL-AFF1 fusion protein in infant acute lymphoblastic leukemia (ALL) (20, 21). It has been well established that fusion of AFF1 to MLL can target SEC to MLL downstream leukemic genes, leading to their misactivation and ultimately leukemogenesis (22). Moreover, recent genome-wide association studies (GWAS) have identified variants in the *AFF1* locus associated with susceptibility to systemic lupus erythematous (23). Missense mutation of *Aff1* in mice leads to ataxia with progressive Purkinje cell loss in the cerebellum (24, 25).

In the present study, we found that high expression of AFF1 significantly correlates with better overall survival in lung cancer patients. AFF1 inhibits NTS transcription in A549 human lung adenocarcinoma cell line, one of the non-small-cell lung cancer (NSCLC) cell types. Chromatin immunoprecipitation sequencing (ChIP-seq) profiling of AFF1 indicated that AFF1 occupies the region located 62 kb downstream from the NTS promoter. CRISPR-cas9-mediated targeted deletion of the AFF1 bound region abolishes NTS transcription. The activity of the AFF1-bound region toward NTS can also be tuned by CRISPR activation or inhibition. Circularized chromosome conformation capture (4C) assay showed that the AFF1-bound region can physically interact with the NTS promoter region, confirming that this region is the NTS enhancer. Thus, AFF1 inhibits NTS transcription through binding its enhancer. Moreover, unsupervised clustering analysis revealed significant positive correlation among the expression of NTS and the cancer promoting factors, such as carbamoyl phosphate synthetase 1 (CPS1) in a subgroup of NSCLC. NTS antagonizes the cytokine interleukin-6 (IL6) pathway to activate CPS1 gene expression. Altogether, our results uncovered the NTS-CPS1 regulatory axis, consisting of AFF1 as the master transcription suppressor and IL6 as the antagonist in lung adenocarcinoma cells.

## Results

### AFF1 occupies the *NTS* downstream region and inhibits *NTS* expression

**Figure 1:**
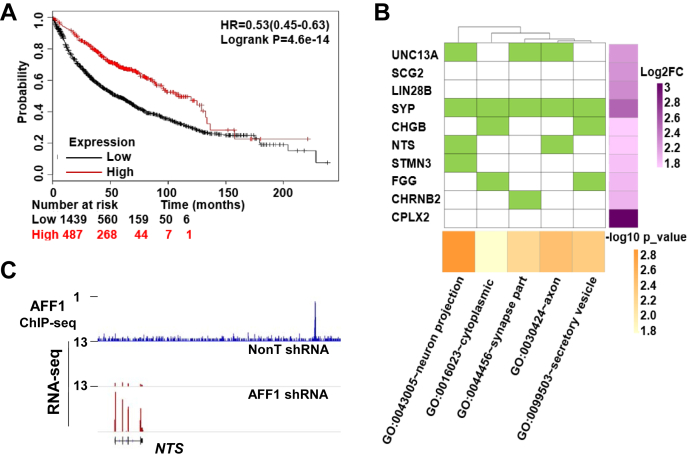
**AFF1 occupies the *NTS* downstream region and inhibits *NTS* expression.***A*, KM plot showing the overall survival of lung cancer patients stratified by AFF1 expression level. *B*, Gene ontology (GO) analysis of the top ten genes that were most upregulated after AFF1 knockdown. *C*, ChIP-seq genome browser track (upper, *blue*) showing the occupancy of AFF1 at the *NTS* downstream region. RNA-seq genome browser track (lower, *purple*) showing increased NTS expression upon AFF1 knockdown.

It has been well established that fusion of AFF1 with MLL is one of the driving forces of infant acute leukemia with poor prognosis (26, 27). In order to explore the possible functions of AFF1 in solid tumors, we first investigated whether AFF1 expression level correlates with the overall clinic outcomes of different cancers. Kaplan–Meier (K-M) plot demonstrated that lung cancer patients with higher expression of *AFF1* showed significant longer survival than those with lower expression of *AFF1* (Fig. 1*A*). To dissect the potential roles of AFF1 in lung cancers, we performed RNA sequencing (RNA-seq) transcriptional profiling studies in human lung adenocarcinoma A549 cells after shRNA-mediated AFF1 knockdown (Fig. S1*A*). Gene ontology (GO) analysis demonstrated that four of the top ten significantly upregulated genes after AFF1 knockdown are involved in neuronal system function, *i.e.*, *NTS* (28), Stathmin 3 (*STMN3*) (29, 30), Synaptophysin (*SYP*) (31, 32), and Unc-13 homolog A (*UNC13A*) (33) (Fig. 1*B*). To understand whether AFF1 directly regulates the expression of these genes, we carried out AFF1 ChIP-seq in A549 cells and found that AFF1 directly occupies the region located 62 kb downstream from the *NTS* promoter (Fig. 1*C*). In contrast, direct binding of AFF1 to the rest three gene loci was not observed (Fig. S1*B*). Thus, AFF1 directly binds to the *NTS* downstream region and suppresses *NTS* expression.

### The AFF1-bound *NTS* downstream region functions as an *NTS* enhancer

**Figure 2:**
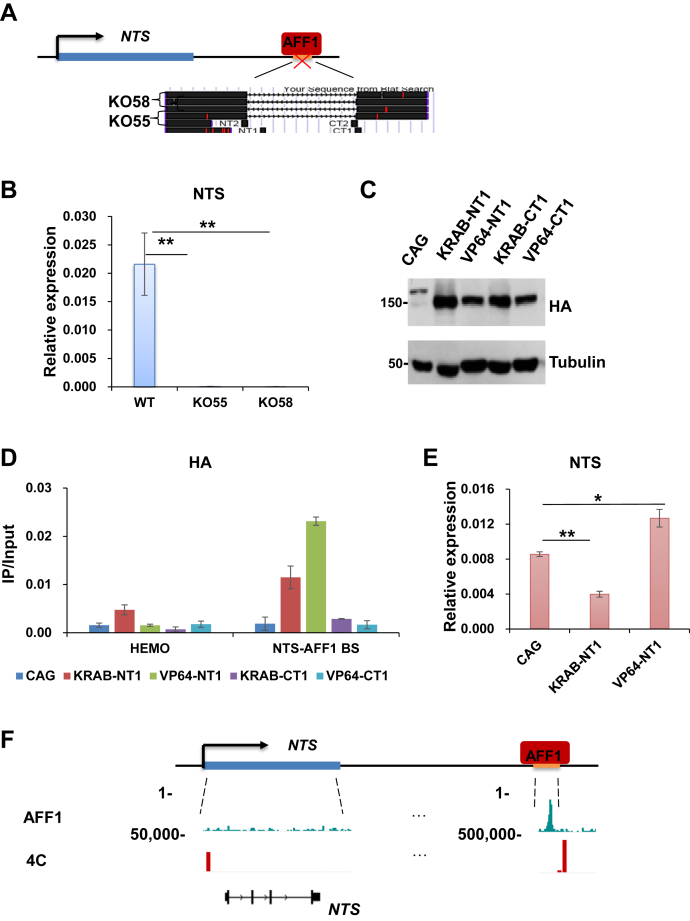
**The AFF1-bound *NTS* downstream region acts as a *NTS* enhancer.***A*, cartoon model illustrating generation of A549 cell lines without the AFF1-bound NTS downstream region. *B*, RT-qPCR showing the expression level of NTS after removal of the AFF1-bound NTS downstream region in A549 cells. Significant differences are marked with an *asterisk* (*t*-test, ∗*p* < 0.05; ∗∗*p* < 0.01; ∗∗∗*p* < 0.001). Error bars represent standard deviations; n = 3. *C*, Western blot showing HA tagged VP64 or KRAB can be detected in A549 cells after lentiviral transduction of the CRISPRa and CRISPRi vectors. *D*, qPCR analysis after HA ChIP showing that specific sgRNAs were able to guide HA tagged VP64 or KRAB to the AFF1-bound NTS downstream region. The *HEMO* gene serves as a negative control for ChIP-qPCR. Error bars represent standard deviations; n = 3. *E*, RT-qPCR showing the expression level of NTS after CRISPR interference and activation of the AFF1-bound NTS downstream region. Significant differences are marked with an *asterisk* (*t*-test, ∗*p* < 0.05; ∗∗*p* < 0.01; ∗∗∗*p* < 0.001). Error bars represent standard deviations; n = 3. (F) 4C-seq showing the physical association between the AFF1-bound NTS downstream region and the *NTS* promoter region.

We asked whether AFF1 can regulate the expression of *NTS* through its binding to the *NTS* downstream region. To test this hypothesis, we first examined the potential activity of this region to *NTS*. CRISPR-mediated genomic deletion was employed to remove the AFF1-bound region from A549 cells by using different sets of short guide RNAs (sgRNAs). Two independent AFF1-bound *NTS* downstream region deleted cell lines were generated (Fig. 2*A*). The expression of *NTS* was almost abolished in the three cell lines, suggesting that this region might possess regulatory activity toward the *NTS* gene (Fig. 2*B*). We then used the CRISPR activation and interference (CRISPRa ad CRISPRi) systems to explore whether the regulatory activity of the AFF1-bound region toward *NTS* can be modulated (34, 35). The fusion of catalytically inactive CRISPR-associated 9 nuclease (dCas9) with the transcription activating effector VP64 or the suppressing effector KRAB can be detected after lentiviral-mediated delivery into A549 cells (Fig. 2*C*). Further analyses showed that dCas9-VP64 and dCas9-KRAB, which can be directed to the AFF1-bound region by specific sgRNA, were capable of tuning up and down *NTS* expression, respectively (Fig. 2, *D* and *E*). To examine whether the AFF1-bound region can interact with *NTS*, we carried out 4C experiment (36, 37) and observed the physical association between this region and the *NTS* promoter region (Fig. 2*F*). Therefore, our results here indicated that the AFF1-bound region is an enhancer that activates the expression of *NTS*, hereinafter referred to as the *NTS* enhancer (*NTS-en*).

### *NTS* is positively correlated with *CPS1*, *FGG*, and *GPX2* expression in a subgroup of NSCLC

**Figure 3:**
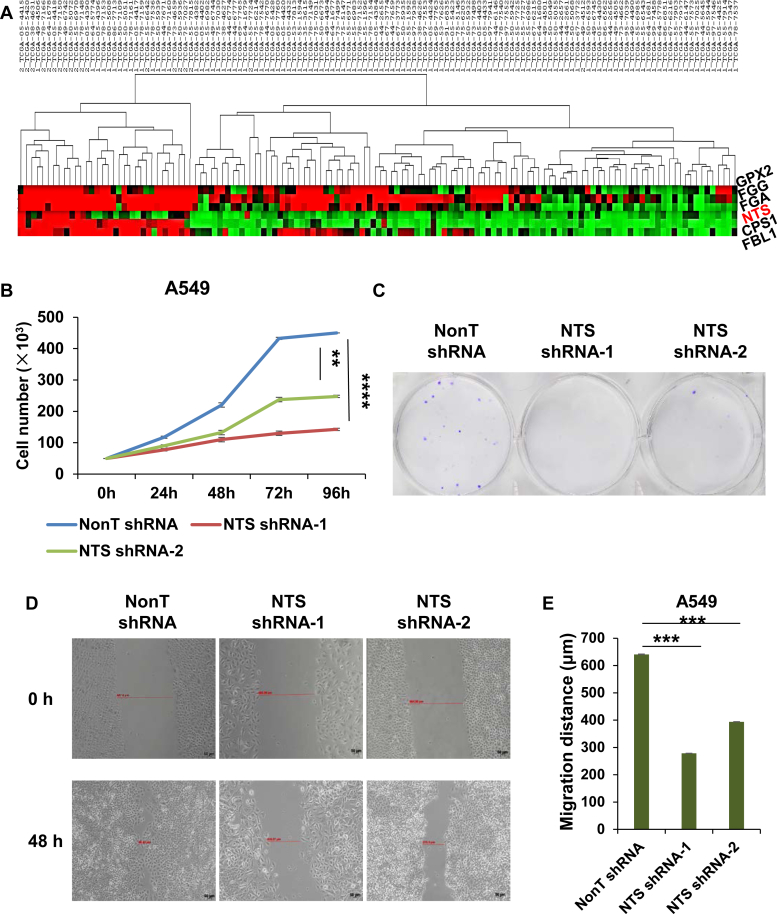
**Positive correlation of *NTS*, *CPS1*, *FGG*, and *GPX2* expression in a subgroup of NSCLC.***A*, unsupervised hierarchical clustering analysis of 133 lung adenocarcinoma patient samples showing positive correlation of NTS and other cancer-promoting factors including CPS1. *B*, nontargeting control or NTS knockdown A549 cells were counted at different time intervals after plating. Statistical significance is marked with an *asterisk* (*t*-test, ∗*p* <0.05; ∗∗*p* <0.01; ∗∗∗*p* <0.001), error bars represent standard deviation, n = 3. *C*, nontargeting control or NTS knockdown A549 cells were stained with crystal violet and imaged. Representative images were showed. *D*, nontargeting control or NTS knockdown A549 cells were imaged at 0 and 48 h after scratches made in the wound healing assay. Representative images were showed. *E*, statistical analysis of cell migration ability after NTS knockdown. Statistical significance is marked with an *asterisk* (*t*-test, ∗*p* <0.05; ∗∗*p* <0.01; ∗∗∗*p* <0.001), error bars represent standard deviation, n = 3.

Upregulation of NTS in a subgroup of NSCLC has been linked to poor prognosis (38, 39). To identify the potential regulatory pathway involving NTS, we performed unsupervised hierarchical clustering analysis of 133 lung adenocarcinoma patient samples to identify genes that share similar expression pattern to NTS. The expression of *NTS* was highly correlated with that of *CPS1*, fibrinogen gamma (*FGG*), and glutathione peroxidase 2 (*GPX2*) in a subgroup of lung adenocarcinoma samples, suggesting these genes might belong to a gene coexpression network (Fig. 3*A*). High expression of *CPS1*, *FGG*, and *GPX2* has been frequently associated with poor prognosis of various cancers (40, 41, 42, 43). It has been reported that CPS1 promotes pyrimidine synthesis in the aggressive subset of lung adenocarcinoma with mutant KRAS plus LKB1 loss and supports tumor growth (44). We found here that NTS knockdown significantly inhibited growth, colony formation, and migration of A549 cells (Fig. 3, *B*–*E*).

### Identification of the AFF1-NTS-CPS1 regulatory axis

**Figure 4:**
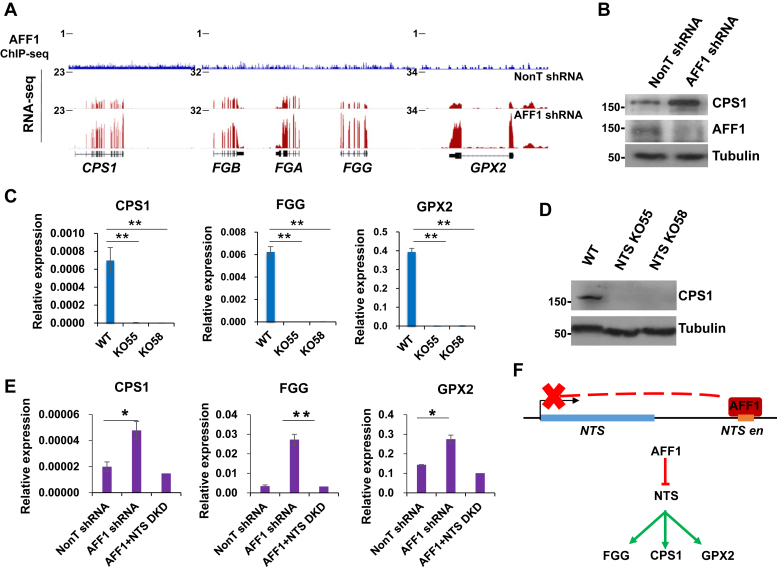
**NTS is required for the proper activation of CPS1, FGG, GPX2 in A549 cells.***A*, ChIP-seq genome browser track (upper, *blue*) showing that AFF1 was not detected at the *CPS1*, *FGG*, and *GPX2* loci. RNA-seq genome browser track (lower, *purple*) showing increased *CPS1*, *FGG*, and *GPX2* expression upon AFF1 knockdown. *B*, Western blot showing that the protein level of CPS1 was increased upon AFF1 knockdown. Tubulin was used as a loading control. *C*, RT-qPCR showing that the RNA levels of CPS1, FGG, and GPX2 were reduced in the *NTS-en* deleted A549 cells. Significant differences are marked with an *asterisk* (*t*-test, ∗*p* < 0.05; ∗∗*p* < 0.01; ∗∗∗*p* < 0.001). Error bars represent standard deviations; n = 3. *D*, Western blot showing that the protein level of CPS1 was reduced in the *NTS-en* deleted A549 cells. Tubulin was used as a loading control. *E*, RT-qPCR showing the expression level of CPS1, FGG, and GPX2 after AFF1 and NTS double knockdown in A549 cells. Significant differences are marked with an *asterisk* (*t*-test, ∗*p* < 0.05; ∗∗*p* < 0.01; ∗∗∗*p* < 0.001). Error bars represent standard deviations; n = 3. *F*, Cartoon model illustrating that AFF1 suppresses NTS expression through binding to *NTS*-*en* (upper) and that AFF1 inhibits the expression of CPS1, FGG, GPX2 *via* NTS (lower).

We noticed that the expression levels of CPS1, FGG, and GPX2, similar to NTS, were also upregulated after AFF1 knockdown (Fig. 4*A*). Upregulation of CPS1 was also validated by western blot (Fig. 4*B*). However, unlike NTS, which was bound by AFF1 at its enhancer, the direct binding of AFF1 to the *CPS1*, *FGG*, and *GPX2* loci was not observed (Fig. 4*A*). Since NTS is the direct AFF1 target gene that is remarkably affected by AFF1 knockdown, we therefore hypothesized that AFF1 might regulate the expression of *CPS1*, *FGG*, and *GPX2 via* NTS. In order to test our hypothesis, we first silenced NTS by shRNA-mediated knockdown in A549 cells. NTS knockdown reduced the expression of *CPS1*, *FGG*, and *GPX2* (Fig. S2*A*). The expression levels of the three factors were also reduced in the *NTS-en* deleted cells, indicating an activating role of NTS in the expression of *CPS1*, *FGG*, and *GPX2* (Fig. 4, *C* and *D*). To further understand whether AFF1 inhibits the expression of the three genes *via* NTS, we performed AFF1 and NTS double knockdown in A549 cells (Fig. S2, *B* and *C*). Quantitative RT-PCR analyses demonstrated that co-knockdown of NTS completely abolished the AFF1 knockdown-induced upregulation of CPS1, FGG, and GPX2, indicating that NTS is required for the suppression of the three genes by AFF1 (Fig. 4, *E* and *F*).

### NTS antagonizes IL6 pathway in A549 cells

In the canonical NTS pathway, NTS binds to its receptors to trigger cellular changes. Three different NTS receptors have been identified to date, namely NTS receptor 1 (NTSR1), NTSR2, and NTSR3 (45, 46). Only NTSR3 can be detected in A549 cells at the RNA level (Fig. S3*A*). However, NTSR3 knockdown led to upregulation of CPS1, FGG, and GPX2, suggesting that activation of the three genes by NTS might be NTS receptor independent (Fig. S3*B*).

**Figure 5:**
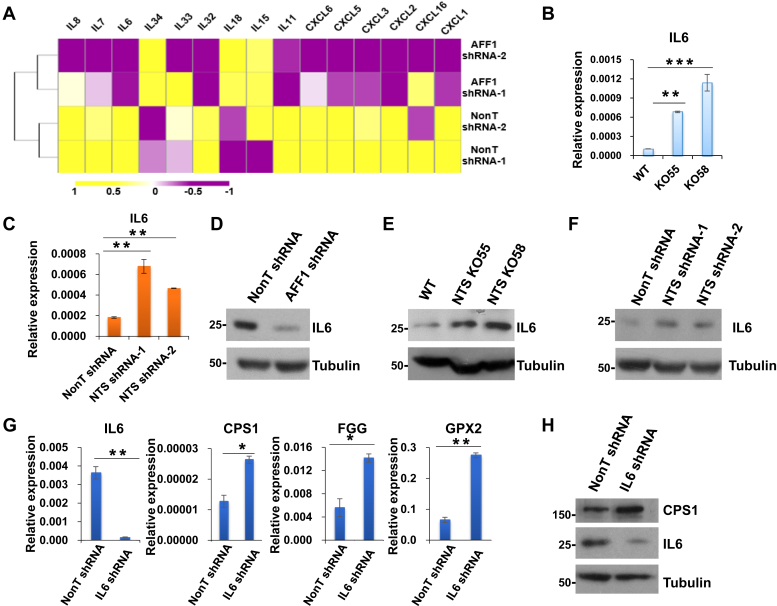
**NTS and IL6 oppositely regulates the expression of CPS1, FGG, and GPX2.***A*, Heatmap showing the expression of several cytokines after AFF1 knockdown in A549 cells. *B*, RT-qPCR showing the expression level of IL6 in the *NTS-en* deleted A549 cells. Significant differences are marked with an *asterisk* (*t*-test, ∗*p* < 0.05; ∗∗*p* < 0.01; ∗∗∗*p* < 0.001). Error bars represent standard deviations; n = 3. *C*, RT-qPCR showing the upregulation of IL6 after NTS knockdown in A549 cells. Significant differences are marked with an *asterisk* (*t*-test, ∗*p* < 0.05; ∗∗*p* < 0.01; ∗∗∗*p* < 0.001). Error bars represent standard deviations; n = 3. D–F, Western blot showing that the protein level of IL6 was reduced upon AFF1 knockdown (*D*), but increased after NTS depletion (*E* and *F*). Tubulin was used as a loading control. *G*, RT-qPCR showing the upregulation of CPS1, FGG, and GPX2 after IL6 knockdown in A549 cells. Significant differences are marked with an *asterisk* (*t*-test, ∗ *p* < 0.05; ∗∗ *p* < 0.01; ∗∗∗ *p* < 0.001). Error bars represent standard deviations; n = 3. *H*, Western blot showing that the protein level of CPS1 was increased upon IL6 knockdown. Tubulin was used as a loading control.

It was reported that NTS could activate interleukin-8 (IL8) to promote local inflammatory and migration of hepatocellular carcinoma (47, 48). Through RNA-seq analyses, we found that a set of cytokines including IL8 and IL6 were differentially expressed after knockdown of the *NTS* upstream regulator AFF1 (Fig. 5*A*). We then examined the levels of several cytokines after *NTS-en* deletion in A549 cells. Quantitative RT-PCR and western blot analyses demonstrated that IL6, instead of IL8, CXCL1, or CXCL5, was significantly upregulated in cells without *NTS-en* (Figs. 5*B* and S4*A*). Consistently, IL6 was activated upon NTS knockdown in A549 cells (Fig. 5*C*). Western blot analyses further indicated that the protein levels of IL6 were regulated by AFF1 and NTS in opposite direction (Fig. 5, *D*–*F*). In order to understand whether IL6 plays a role in NTS-mediated gene expression regulation, we performed shRNA-mediated IL6 knockdown in A549 cells. The expression levels of CPS1, FGG, and GPX2 were increased upon IL6 knockdown, indicating that NTS and IL6 oppositely regulate the expression of the three genes (Fig. 5, *G* and *H*). In addition, we found that knockdown of the IL6 signal transducer IL6ST also led to increases in the RNA levels of CPS1, FGG, and GPX2, further substantiating the role of the IL6 pathway in antagonizing NTS (Fig. S4*B*) (49, 50).

## Discussion

The small neuropeptide NTS is significantly activated in a subgroup of NSCLC and associated with poor prognosis. However, the regulatory axis involving NTS in NSCLC remains unclear. In the present study, we identified the NTS-specific enhancer, which is located 62 kb downstream from the NTS promoter. AFF1 occupies the NTS enhancer and suppresses NTS transcription. NTS is highly positively correlated with the expression of the cancer-promoting factors CPS1, FGG, and GPX2 in a subgroup of NSCLC. Detailed analyses demonstrated that NTS antagonizes the IL6 pathway in regulating the CPS1, FGG, and GPX2. Thus, our analyses revealed a novel NTS-centered regulatory axis, consisting of AFF1 as a master transcription regulator and IL6 as an antagonist in a subgroup of NSCLC.

It has been shown previously that some of the transcription elongation factors can regulate transcription through enhancer (51, 52, 53). We have previously shown that ELL3 occupies inactive or repressed enhancers and regulates the establishment of paused Pol II at nearby genes in mouse embryonic stem cells (51). ELL3 also functions as a platform in promoting SEC assembly at promoters during stem cell differentiation. AFF1, the core component of SEC, has been well known for its function in regulating transcriptional pause and release (54, 55). The present study demonstrates a role of AFF1 in suppressing gene expression through binding to enhancer. Although the precise mechanism through which AFF1 mediates gene suppression from enhancer has not been clearly elucidated, it is in line with the previous studies showing that AFF3, the other member of the AFF family, is able to occupy both active and inactive cis-regulatory elements to regulate the expression of *XIST* and the imprinted genes *via* different zinc finger transcription factors (56, 57, 58).

NTS, acting as a signal transmitter, has been shown to cause sustained activation of epidermal growth factor receptor (EGFR) and enhanced cancer growth (8, 59). CPS1, one of the NTS downstream factors identified in the current study, is the rate-limiting enzyme in urea cycle and promotes proliferation and tumor growth (60). High level of CPS1 in NSCLC is correlated with poor prognosis. It has been found that the growth of EGFR-driven NSCLC relies on CPS1-mediated urea cycle. EGFR inhibition, once combined with CPS1 knockdown, could further reduce proliferation of NSCLC (61). Here we found that NTS is the upstream activator for CPS1 expression. As the upstream regulator of both EGFR and CPS1, NTS could be a potential therapeutic target in the treatment of the subgroup of NSCLC with high expression of all the three factors.

## Experimental procedures

### Cell culture

A549 and HEK293T cells were cultured in Dulbecco's modified eagle medium (Sigma) containing 10% fetal bovine serum (Ex-Cell Bio) and 1% Penicillin-Streptomycin (HyClone) at 37 °C with 5% CO_2_ in a cell incubator.

### Antibodies

Antibody against AFF1 for ChIP-seq was previously described (15). Antibody against AFF1 (A302-344A) was purchased from Bethyl. Antibodies against IL6 (A0286) and CPS1 (A8080) were purchased from Abclonal. Antibody against HA (sc-805) was purchased from Santa Cruz.

### Lentivirus-mediated RNA interference (RNAi)

Human AFF1, NTS, NTSR3, IL6, and IL6ST shRNA were cloned into the pLKO.1 vector (Addgene #10787). HEK293T cells were plated in 90 mm culture plate and cotransfected with 4 μg shRNA construct or nontargeting shRNA construct, 3 μg psPAX2 packaging plasmid, and 1 μg pMD2.G envelope plasmid using polyethyleneimine (Sigma). Lentiviral supernatants were collected 48 and 72 h after transfection and filtered using 0.45 μm filters. A549 cells were infected with the filtered lentiviral supernatants with polybrene (Sigma) at the concentration of 8 μg/ml. After 24 h, 2 μg/ml of puromycin (Invitrogen) was used to select the infected cells.

### CRISPRi and CRISPRa

sgRNA oligos targeting the AFF1-bound *NTS* downstream region were cloned into the pX459 (Addgene #48139). U6 promoter-sgRNA fragments were amplified and cloned into the pHAGE EF1ɑ dCas9-KRAB (Addgene #50919) and pHAGE EF1ɑ dCas9-VP64 (Addgene #50918). Lentiviral particle preparation and infection were performed as described above.

### CRISPR-Cas9-guided knockout

sgRNA oligos targeting the AFF1-bound *NTS* downstream region were cloned into the lentiCRISPR v2 (Addgene #52921). A549 cells were infected and selected with puromycin for 48 h. The infected cells were maintained until cell clones were ready to be picked. The clones were screened with polymerase chain reaction (PCR) and confirmed by T-vector cloning and sequencing.

### Western blot

Whole-cell lysates were prepared and loaded onto SDS-polyacrylamide gel electrophoresis gels. Proteins were transferred to polyvinylidene fluoride membranes. Primary antibodies used were incubated overnight at 4 °C. HRP-conjugated secondary antibodies (Sigma) were used at a dilution of 1:5000. enhanced chemiluminescence substrate (Millipore) was applied to the membrane for imaging by autoradiography.

### Quantitative RT-PCR and RNA-seq library preparation

Total RNA was isolated using the total RNA kit (Qiagen) according to the manufacturer's protocol, treated with RNase-free DNase Ⅰ (NEB), and repurified with RNA kit. cDNA was generated using RT reagent mix. Real-time qPCR was performed using SYBR Green mix (Yeasen) on Bio-Rad CFX96-Real Time System. The relative expression levels of genes of interest were normalized to the expression of the housekeeping gene *GAPDH*. Relative fold changes in gene expression were calculated using the ΔΔCT method. For RNA-seq library preparation, polyadenylated RNA was purified from total RNA and fragmented. Double-stranded cDNA from the RNA fragments was ligated to adapters before being subjected to deep sequencing (Illumina).

### ChIP and ChIP-seq library preparation

In total, 5 × 10^7^ A549 cells were used per ChIP or ChIP-seq assay. Cells were cross-linked in phosphate buffered saline containing 1% formaldehyde to the cell culture media at room temperature for 10 min, and cross-linking was quenched by glycine. Fixed chromatin was sonicated into 200–800 base pair fragments (Bioruptor, Diagenode) in chromatin immunoprecipitation (ChIP) lysis buffer 10 mM tris-HCl [pH 8.0], 100 mM NaCl, 1 mM EDTA, 0.5 mM EGTA, 0.1% Na-deoxycholate, and 0.5% N-lauroylsarcosine supplemented with protease inhibitor cocktail (Sigma). Chromatin extracts were incubated with AFF1 antibody and protein A agarose beads at 4 °C overnight. Immunoprecipitates were washed with radio immunoprecipitation assay buffer (50 mM Hepes-KOH [pKa 7.55], 500 mM LiCl, 1 mM EDTA, 1.0% NP-40, and 0.7% Na-deoxycholate) for five times and TE once. After the final wash, DNA was eluted and reverse cross-linked at 65 °C. DNA was then purified and used as a template for qPCR. For ChIP-seq, libraries were prepared with Illumina's ChIP seq sample prep kit.

### Circular chromosome conformation capture sequencing (4C-seq)

In total, 1 × 10^7^ A549 cells were used per 4C assay. Cells are treated with formaldehyde, which cross-links proteins to proteins and DNA. Cross-linked chromatin is subsequently digested with HindIII (NEB). Next, chromatin is diluted and then religated by T4 ligase (NEB) to fuse the ends of DNA fragments. DNA is further digested by DpnII (NEB) that digests the fragment into smaller fragments after the removal of cross-links by heating. These fragments are religated under diluted conditions to create much smaller DNA circles using T4 ligase (NEB) again. Inverse PCR primers with Illumina forward and reverse adaptors were designed to anneal to a bait locus HindIII/DpnII restriction fragment. A total of 3200 ng of 4C template was used to amplify each bait using Expand Long Template Polymerase (Roche) system. The PCR program is as follows: 2 min at 94 °C; 10 s at 94 °C; 1 min at bait specific annealing temperature; 3 min at 68 °C; 29 × repeat; 5 min at 68 °C; hold at 4 °C. All 16 PCR tubes were pooled and purified using High Pure PCR Product Purification Kit (Roche) kit. Subsequently, high-throughput sequencing is used to detect the captured regions.

### Cluster analysis

An open-source clustering software cluster3.0 was used for hierarchical clustering analysis, and Java Tree View was used for cluster visualization. Hierarchical clustering analysis of the expression of *NTS* and the genes that showed a strong correlation for NTS (FDR < 0.01) was performed. Data set: The Cancer Genome Atlas Lung Adenocarcinoma (TCGA-LUAD) data.

### Colony formation analysis

Nontargeting control or NTS knockdown A549 cells were digested, counted. In total, 1000 cells were plated with each well of a six-well plate. After 2 weeks of maintenance, cells were stained with crystal violet staining solution (Sangon) and photographed and stored. Each set of experiments was repeated three times.

### Cell proliferation analysis

Nontargeting control or NTS knockdown A549 cells were digested, counted. In total, 4 × 10^5^ cells were plated with each well of a six-well plate. Cells were harvested every 24 h for cell counting until the cells reached 100% confluence.

### Wound healing assay

Nontargeting control or NTS knockdown A549 cells were digested, counted. In total, 5 × 10^5^ cells were cultured with each well of a six-well plate for 24 h. A straight scratch was made on the monolayer of the cells using a P200 pipette tip. Cells were then washed with 1 × phosphate buffered saline for three times to remove the floating cells. The cells were imaged, and the gap width was measured at different time intervals.

## Data availability

RNA-seq after AFF1 knockdown and AFF1 ChIP-seq data sets are available from the GEO database under accession number GSE164098.

## Conflict of interest

The authors declare that they have no conflicts of interest with the contents of this article.
