# Supplementary material for: Suppression of the NTS-CPS1 regulatory axis by AFF1 in lung adenocarcinoma cells
Source: J Biol Chem. 2021 Jan 22;296:100319. doi: 10.1016/j.jbc.2021.100319 (PMC7949158; doi:10.1016/j.jbc.2021.100319)
Supplement: Figures S1 to S4 [file mmc1.pdf]

Supplementary Figure Legends

Figure S1. AFF1 inhibits *SYP, STMN3* and *UNC13A* expression. (A) RNA-seq genome browser track showing AFF1 knockdown efficiency. (B) ChIP-seq genome browser track (upper, blue) showing that AFF1 was not detected at the *SYP*, *STMN3* and *UNC13A* loci. RNA-seq genome browser track (lower, purple) showing increased *SYP*, *STMN3* and *UNC13A* expression upon AFF1 knockdown.

Figure S2. AFF1 inhibits the expression of CPS1, FGG, GPX2 via NTS. (A) RT-qPCR showing that the RNA levels of CPS1, FGG and GPX2 were reduced after NTS knockdown in A549 cells. Significant differences are marked with an asterisk (t-test, * p < 0.05; ** p < 0.01; *** p < 0.001). Error bars represent standard deviations; n=3; (B-C) RT-qPCR showing the knockdown efficiency of AFF1 and NTS double knockdown in A549 cells. Significant differences are marked with an asterisk (t-test, * p < 0.05; ** p < 0.01; *** p < 0.001). Error bars represent standard deviations; n=3.

Figure S3. The activation of CPS1, FGG and GPX2 by NTS might be NTS receptor independent. (A) RNA-seq genome browser track showing the expression levels of NTSR1, NTSR2 and NTSR3 in A549 cells. (B). RT-qPCR showing the expression levels of CPS1, FGG and GPX2 after NTSR3 knockdown in A549 cells. Significant differences are marked with an asterisk (t-test, * p < 0.05; ** p < 0.01; *** p < 0.001). Error bars represent standard deviations; n=3.

Figure S4. IL6ST inhibits the expression of CPS1, FGG and GPX2. (A) RT-qPCR showing the expression level of IL8, CXCL1 and CXCL5 in the *NTS-en* deleted A549 cells. Error bars represent standard deviations; n=3. (B) RT-qPCR showing the up-regulation of CPS1, FGG and GPX2 after IL6ST knockdown in A549 cells. Significant differences are marked with an asterisk (t-test, * p < 0.05; ** p < 0.01; *** p < 0.001). Error bars represent standard deviations; n=3.

FIG.S1

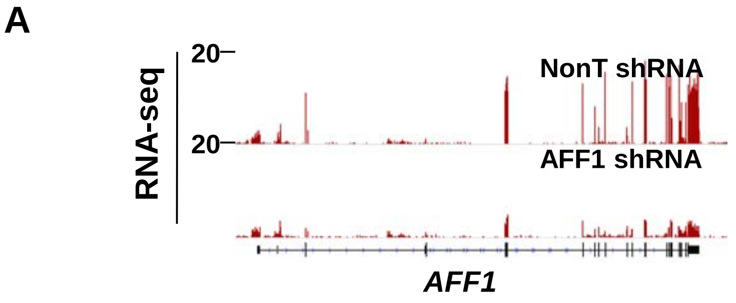

A
RNA-seq
20
NonT shRNA
20
AFF1 shRNA
AFF1

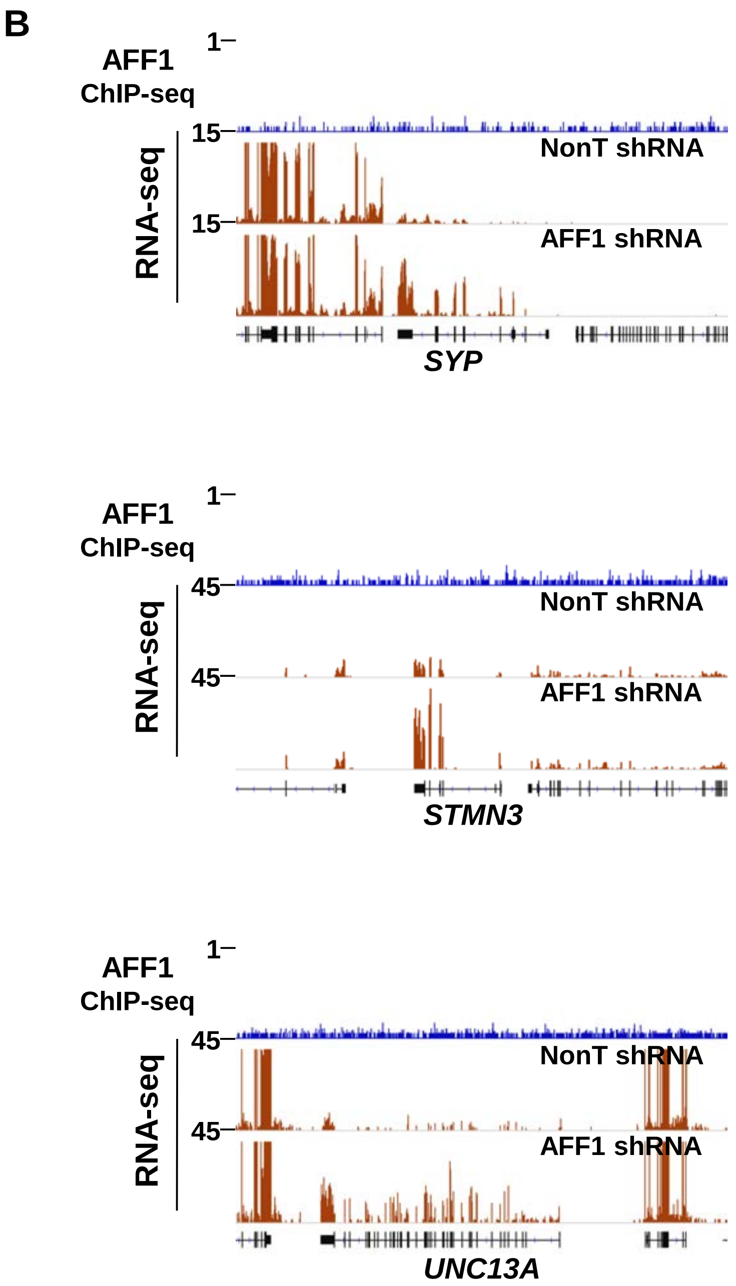

B
AFF1
ChIP-seq
1
RNA-seq
15
NonT shRNA
15
AFF1 shRNA
SYP
AFF1
ChIP-seq
1
RNA-seq
45
NonT shRNA
45
AFF1 shRNA
STMN3
AFF1
ChIP-seq
1
RNA-seq
45
NonT shRNA
45
AFF1 shRNA
UNC13A

FIG.S2

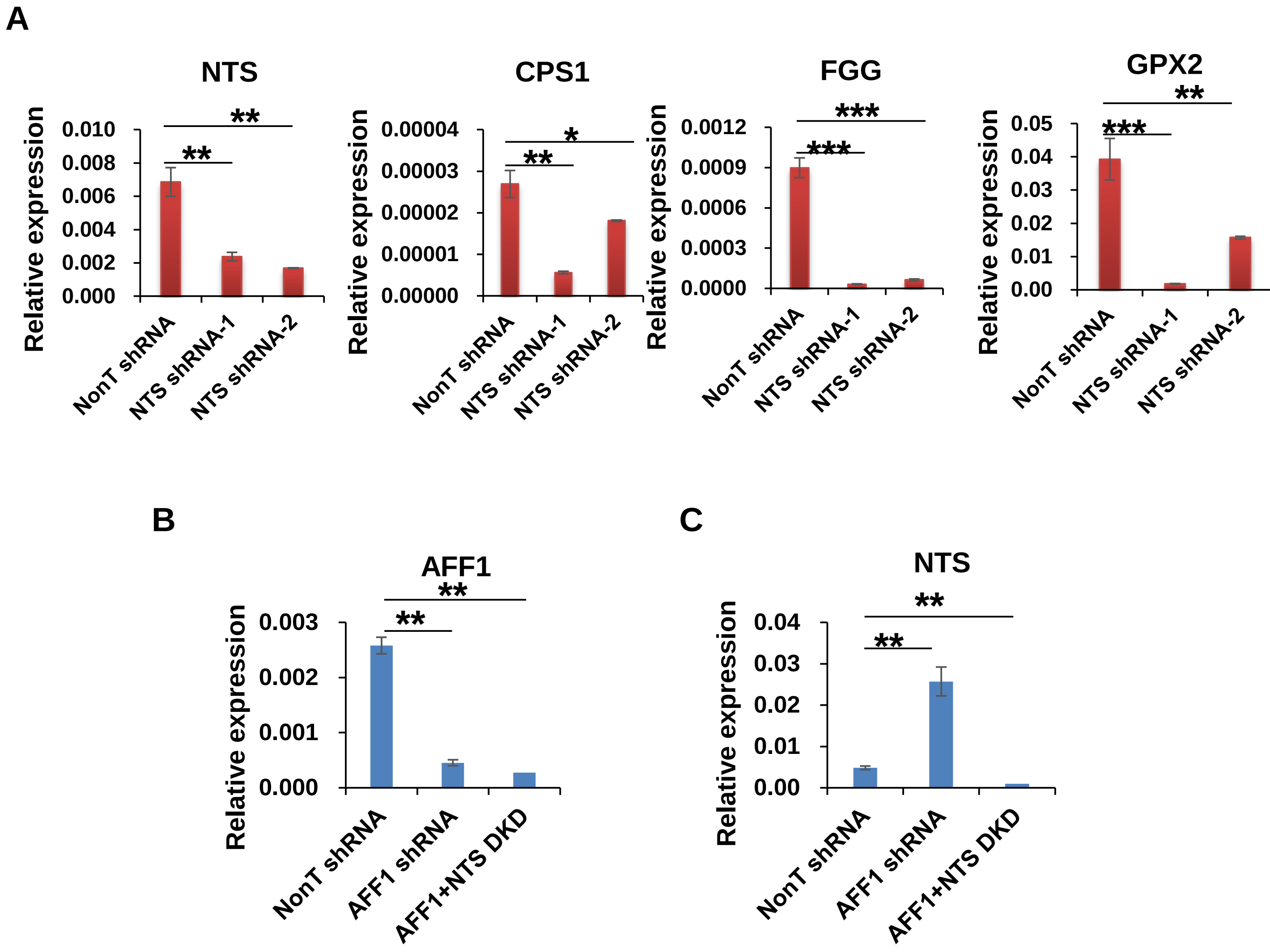
A
NTS
Relative expression
0.010
0.008
0.006
0.004
0.002
0.000
**
**
NonT shRNA
NTS shRNA-1
NTS shRNA-2
CPS1
Relative expression
0.00004
0.00003
0.00002
0.00001
0.00000
*
**
NonT shRNA
NTS shRNA-1
NTS shRNA-2
FGG
Relative expression
0.0012
0.0009
0.0006
0.0003
0.0000
***
***
NonT shRNA
NTS shRNA-1
NTS shRNA-2
GPX2
Relative expression
0.05
0.04
0.03
0.02
0.01
0.00
**
***
NonT shRNA
NTS shRNA-1
NTS shRNA-2
B
AFF1
Relative expression
0.003
0.002
0.001
0.000
**
**
NonT shRNA
AFF1 shRNA
AFF1+NTS DKD
C
NTS
Relative expression
0.04
0.03
0.02
0.01
0.00
**
**
NonT shRNA
AFF1 shRNA
AFF1+NTS DKD

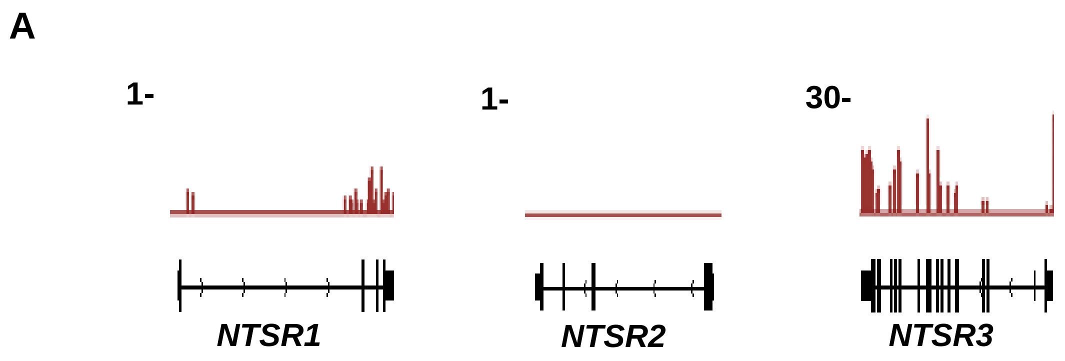
A
1-
1-
30-
NTSR1
NTSR2
NTSR3

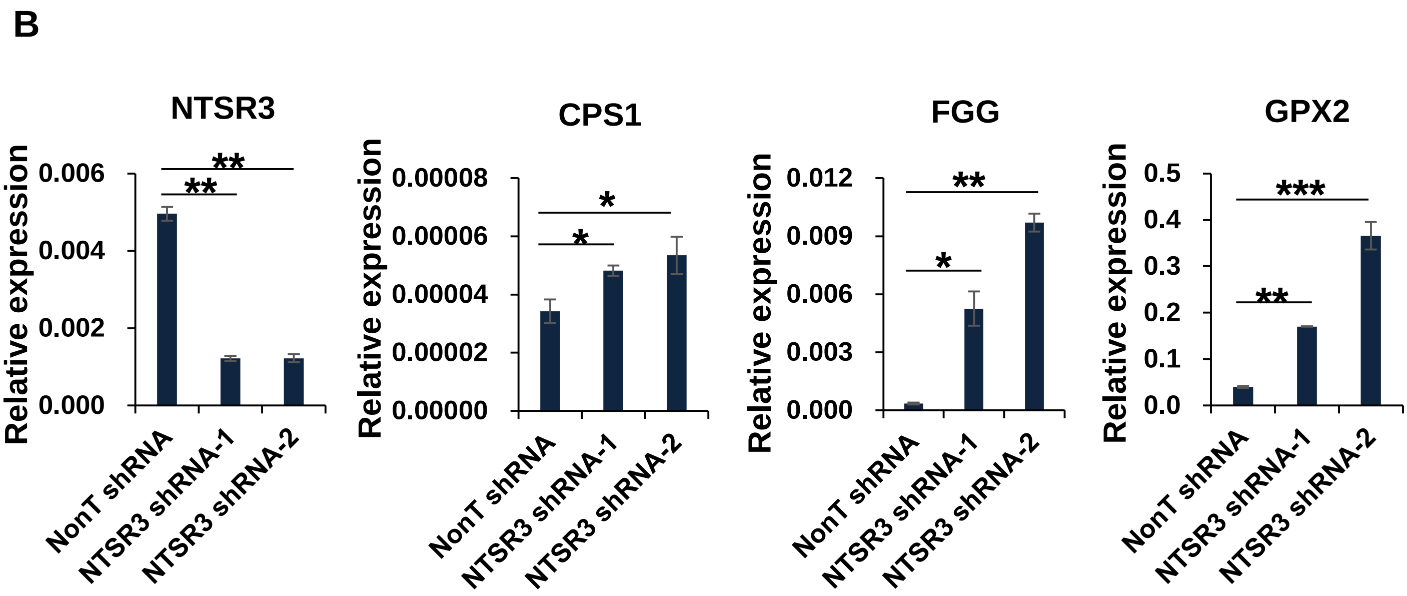
B
NTSR3
CPS1
FGG
GPX2
Relative expression
0.006
0.004
0.002
0.000
0.00008
0.00006
0.00004
0.00002
0.00000
0.012
0.009
0.006
0.003
0.000
0.5
0.4
0.3
0.2
0.1
0.0
NonT shRNA
NTSR3 shRNA-1
NTSR3 shRNA-2
*
**
***

FIG.S4

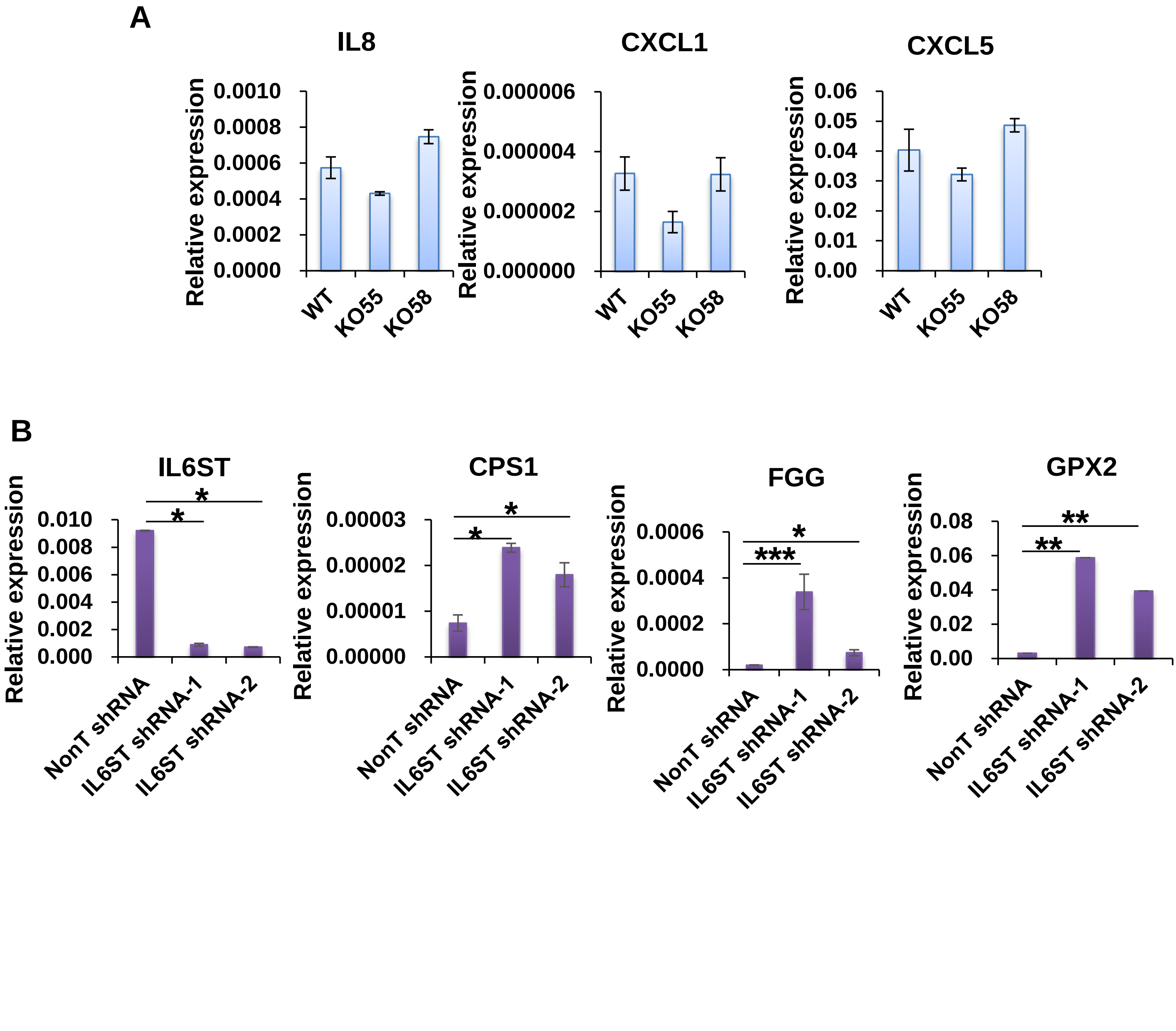
A
IL8
CXCL1
CXCL5
Relative expression
0.0010
0.0008
0.0006
0.0004
0.0002
0.0000
0.000006
0.000004
0.000002
0.000000
0.06
0.05
0.04
0.03
0.02
0.01
0.00
WT
KO55
KO58
B
IL6ST
CPS1
FGG
GPX2
0.010
0.008
0.006
0.004
0.002
0.000
0.00003
0.00002
0.00001
0.00000
0.0006
0.0004
0.0002
0.0000
0.08
0.06
0.04
0.02
0.00
*
**
***
NonT shRNA
IL6ST shRNA-1
IL6ST shRNA-2
